# Scanning Photocurrent Microscopy in Single Crystal Multidimensional Hybrid Lead Bromide Perovskites

**DOI:** 10.3390/nano13182570

**Published:** 2023-09-16

**Authors:** Elena Segura-Sanchis, Rocío García-Aboal, Roberto Fenollosa, Fernando Ramiro-Manzano, Pedro Atienzar

**Affiliations:** Instituto de Tecnología Química, Consejo Superior de Investigaciones Científicas, Universitat Politècnica de València, Avenida de los Naranjos s/n, 46022 Valencia, Spain; elsesan@itq.upv.es (E.S.-S.); rociogarciaaboal@gmail.com (R.G.-A.); rfenollo@ter.upv.es (R.F.)

**Keywords:** perovskite, diffusion length, multidimensional, dynamic, SPCM, single crystal

## Abstract

We investigated solution-grown single crystals of multidimensional 2D–3D hybrid lead bromide perovskites using spatially resolved photocurrent and photoluminescence. Scanning photocurrent microscopy (SPCM) measurements where the electrodes consisted of a dip probe contact and a back contact. The crystals revealed significant differences between 3D and multidimensional 2D–3D perovskites under biased detection, not only in terms of photocarrier decay length values but also in the spatial dynamics across the crystal. In general, the photocurrent maps indicate that the closer the border proximity, the shorter the effective decay length, thus suggesting a determinant role of the border recombination centers in monocrystalline samples. In this case, multidimensional 2D–3D perovskites exhibited a simple fitting model consisting of a single exponential, while 3D perovskites demonstrated two distinct charge carrier migration dynamics within the crystal: fast and slow. Although the first one matches that of the 2D–3D perovskite, the long decay of the 3D sample exhibits a value two orders of magnitude larger. This difference could be attributed to the presence of interlayer screening and a larger exciton binding energy of the multidimensional 2D–3D perovskites with respect to their 3D counterparts.

## 1. Introduction

Hybrid halide perovskites are highly promising materials, not only for the next-generation photovoltaics but also in many other fields where optoelectronics plays a key role. These applications include light-emitting diodes (LEDs), photodetectors, lasers, sensors, and field-effect transistors [1]. Their tunable bandgap, defect tolerance, long carrier diffusion length and high carrier mobility are noteworthy. In addition, they can be easily processed with a low fabrication cost. On the other hand, the huge amount of possibilities that these materials offer in terms of composition [2], morphology and synthetic methods makes a deep understanding of their intrinsic properties mandatory in order to know their potential advantages in commercial applications. 

Typically, hybrid perovskites are based on a structural base such as ABX_3_ in the three dimensions, where A and B correspond to monovalent and divalent cations, respectively, with different sizes, and X are halides. However, these perovskites have certain limitations, mainly related to the stability to moisture. The reduction of the perovskite dimension in the form of a 2D layered structure, analogous to a conventional van der Waals material, has been shown to attenuate such an undesirable effect by providing hydrophobic shielding. This is achieved, for instance, by introducing a large organic cation (M) between [PbX_6_]^4−^ layers as a spacer. In addition, 2D structures yield other effects related to quantum confinement, band alignment, passivation of trap states and inhibition of ion movement [3,4,5]. On the other hand, the quantum confinement confers a strong exciton binding energy, which hinders their suitability for use in solar cells because the free carrier generation is significantly suppressed. In this regard, multidimensional perovskites that combine 2D–3D structures have proven to be a promising alternative, because they represent a tradeoff between stability and performance. The general formula of these perovskites is M_2(n)_A_n−1_B_n_X_3n+1_, where *n* represents the number of metal halide interlayers, estimated from the stichometry of the precursors [6,7]. Therefore, the *n* value defines the bulk-like block and thus the 3D-like behavior inside the van der Waals stacking.

One of the most important parameters employed in the evaluation of various perovskite structures and compositions in terms of charge transport is the diffusion length [8]. This parameter corresponds to the average distance an excited charge carrier can travel towards a collecting electrode before recombining through mechanisms such as radiative recombination or trap-assisted recombination [9]. It has already been demonstrated that large single crystals of perovskites containing iodine in their structure can achieve diffusion lengths ranging from several to even thousands of micrometers [10,11]. Furthermore, hybrid perovskite materials containing iodide ions in their structure have been observed to exhibit longer electron and hole diffusion lengths compared to bromide perovskites [12]. However, it is worth noting that MAPbBr_3_ perovskite materials are significantly more stable under ambient conditions than MAPbI_3_ perovskite materials [13], making this composition preferable for studying intrinsic electronic properties. The introduction of a large cation tends to reduce the diffusion length of the photogenerated carriers, although it remains within the same order of magnitude as that of 3D perovskites [10]. However, different values have been reported in the literature and depend on the experimental tool used for evaluating it. For instance, optical probing methods based on photoluminescence or transient absorption do not monitor all the possible charges produced, such as those thermally emitted from traps states [14]. Furthermore, various types of charge carrier transport have been studied. For example, Guo et al. [14] proposed different transport regimes, including quasiballistic transport where charge carriers can travel long distances without significant scattering or collisions, nonequilibrium transport depending on conditions and energy input, and diffusive transport characterized by random scattering events, leading to a random walk-like behavior [14,15]. Keeping in mind that the transport is strongly influenced by defects and the grain boundaries the interfaces present in polycrystalline films could be a determinant in their photocarrier response. Furthermore, the generation of hot carriers with excess energy, as well as the formation of excitons and free charges in hybrid perovskites, have implications for the transport processes [16].

A versatile technique for studying the carriers’ generation and transport in semiconductors during the excitation is the use of a scanning confocal photocurrent microscopy (SCPM) [17,18,19,20,21,22,23], which allows the current generated in different regions of the sample by optical excitation to be mapped. Additionally, fluorescence information can be simultaneously acquired, which makes it possible to analyze the radiative processes of the samples being studied. This technique has been widely employed to investigate the morphology and photocurrent generation in a wide range of semiconducting materials, including silicon [24], cadmium telluride [25], lead selenide [26], organic heterojunctions [27] and others [28]. In this context, we developed a confocal microscopy tool that allowed us to demonstrate the behavior of hybrid perovskites and octahedral molybdenum clusters as Fabry–Pérot cavities [29,30], as well as the behavior of perovskite nanowires as photonic waveguides [31], and to measure the enhanced photoresponse of perovskites containing subphthalocyanines [32]. In all the cases, the studies were realized on micrometer-size single crystals because they allow perturbing factors which are present in a poly-crystalline sample such as the grain boundaries to be disregarded. Parameters such as size, shape, crystal orientation and composition can have a significant influence, but generally, single crystals exhibit much better optoelectronic properties than their polycrystalline counterparts [33,34].

**Figure 1:**
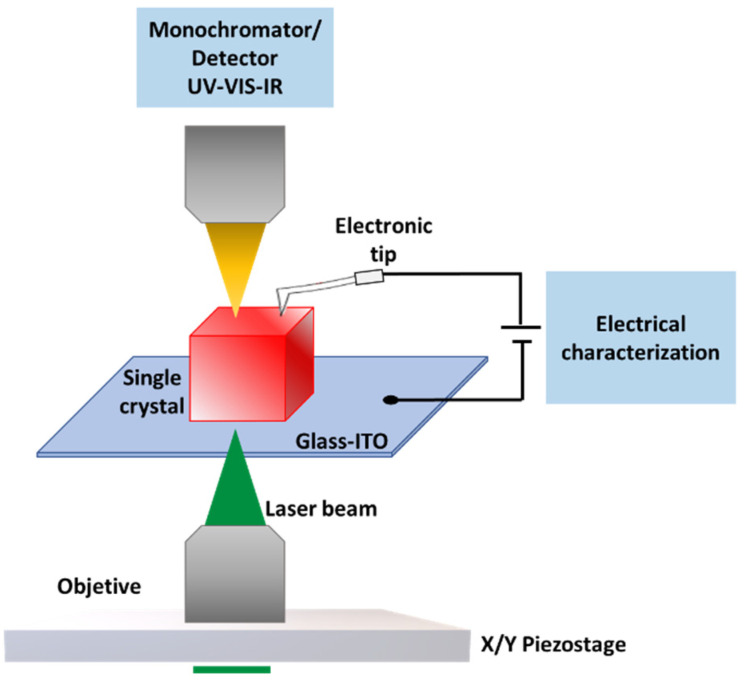
Schematic representation of the electric contact on the microcrystalline samples, showing the irradiation objective at the bottom part controlled with a piezoelectric stage.

In this work, we take a step further by introducing a scanning excitation system and a punctual electrical probe that can be positioned freely on the sample area with micrometer precision in a top (and back) contact configuration (Figure 1). This approach is similar to the widely used technique of SCPM (all back contact) that allows estimating the photocarrier decay lengths under a certain bias. This decay length corresponds to the diffusion length (*L_d_*) evaluating the sample region without the influence of the external electric field or under low bias [35,36]. This method offers advantages over other types of experiments where *L_D_* is deduced from a combination of carrier diffusion (D) and lifetime measurements (*τ*), ($L_{d}=\sqrt{D\tau }$).

There are some examples reported in the literature that study the diffusion length over single crystals using an SCPM system. For instance, Shreetu et al. demonstrated on millimeter-sized crystals that hybrid perovskites having iodine in the structure show a long in-plane charge carrier diffusion length of above 7–15 μm depending on the n value in the structure [10]. Regarding single crystals of perovskites containing bromide halide in their structure, Ganesh et al. reported diffusion length values of 13.3 μm and 13.8 μm for electrons and holes, respectively [37]. In addition, Zhang et al. studied the role of chlorine incorporation in the perovskite structure, resulting in diffusion lengths of several hundreds of micrometers [38]. In any case, the direct extraction of *L_d_* requires several conditions to be fulfilled. For a 1D system, *L_d_* must be much larger than the cross section and the distance between contacts is chosen to be larger than *L_d_* [39] or the region to characterize is placed out of the electrode gap [35]. In this way, *L_d_* from lithographically patterned strips, synthetized nanotubes or fibers could be extracted. In addition, other effects or parameters such as the influence of a bias voltage, the charge drift length, or the determination of the minority carriers have being studied [35]. In principle, this single exponential decay model of *L_d_* could be directly extended to planar schemes by changing the electrode shape from an ideal single point to an infinite line [28]. This is a difficult condition to fulfill. However, assuming that the sample thickness is smaller than *L_d_*, it has been proven that *L_d_* could be extracted (employing a single exponential decay) regardless of the width of the planar sample [40]. On the other hand, this technique permits evaluating the inhomogeneity of different sections when a back-contact planar scheme is used [36]. In fact, it simplifies the study methodology because the sample is directly sensitized on the electrodes, formed by transparent conductive contacts with micrometer gaps, thus eliminating the need for a sample transfer process [35].

Herein, we present a comparative study of the photocurrent decay length in 3D and multidimensional 2D–3D perovskites planar micrometer-size single crystals that have bromide in their structure. Our aim is to compare various azimuth sections, focusing particularly on their possible dependency with the outer border distance. Our scheme does not fulfill the requirements of the single exponential *L_d_* extraction, because the contact is a mixture of a point contact and a planar surface and our sample thickness (tens of µm) surpass *L_d_* values (for the case of the multidimensional sample) in the literature; thus, it could not be assumed to be a 2D sample/measurement scheme. Nevertheless, our point probe scheme makes it possible to measure the photocurrent decay at various azimuthal angles. Since the *L_d_* conditions are not fulfilled, a certain bias has been chosen (−1.25V) to acquire low-noise signals from samples with low photocurrent efficiency, such as multidimensional 2D–3D perovskites.

## 2. Experiment

### 2.1. Perovskite Synthesis

For the preparation of the single crystal hybrid perovskite, the antisolvent method was employed. Firstly, this method consists of dissolving the precursors in a small amount of DMF (1 mL). For 3D perovskites (MAPbBr_3_), the composition consisted of 0.5 M methyl ammonium bromide (MABr) and 0.5 M lead bromide (PbBr_2_), while for the 2D–3D perovskite ((PEA)_2_(MA)_n−1_Pb_n_Br_3n+1_, n = 10), it was 0.4 M MABr, 0.5 M PbBr_2_ and 0.2 M phenylethyl ammonium bromide (PEABr). This mixture (20 µL) was placed on a conductive substrate (ITO on glass, supplied by Ossila (Sheffield, UK)) on a Teflon stage inside a sealed vial, and THF was used as the antisolvent (5 mL) (see Figure S6). The vessel was completely sealed and kept in the dark until small orange-pinkish crystals were observed. At least 24 h are required to obtain good-quality crystals larger than 20 µm. The crystals were manually selected using Gel-Pack (Hayward, CA, USA) and a micromanipulator Narishige (Tokyo, Japan) MMN-1 coupled to a Nikon (Tokyo, Japan) Eclipse LV100 microscope to perform the corresponding characterization. MABr and PbBr_2_ were purchased from ABCR (Karlsruhe, Germany). THF and DMF were purchased from Sigma-Aldrich (St. Luis, MO, USA) and PEABr.

### 2.2. Experimental Set-Up for SCPC Measurements

With the purpose of studying the photoresponse of both 3D and multidimensional 2D–3D perovskites, we placed a selected crystal onto a glass substrate coated with indium-tin oxide (ITO), which served as the back-contact electrode (Figure 1). Then an electronic probe was positioned on the crystal. This established the top contact. The light of a laser with λ = 405 nm and P = 350 µW was chopped and focused onto the sample through the back-contact transparent electrode by means of a microscope objective that was mounted on a piezo system. The photocurrent signal from the sample was acquired with a lock-in amplifier through a transimpedance amplifier that provides the bias voltage as well. The photocurrent response was mapped by varying the objective position with the help of the piezo system. At the same time, the photoluminescence signal was acquired employing a photodiode connected to a second lock-in amplifier. This measurement makes it possible to check the crystal stability condition. With the aim of limiting the sample degradation and minimizing possible fluctuations in the response [41], the scanning time was minimized by setting the position control of the piezo system to the open-loop mode. In this case, the signals that drive the piezo actuator are not continuously corrected according to the sensed position (closed-loop scheme). As a result, the elapsed time for the characterization (scanning area of 80 µm × 80 µm) is reduced about three times, from 791.7 s (open loop) to 243.2 s (closed loop), acquiring only the forward scan direction of zig-zag paths. Despite the non-linear response of the piezo system, which may introduce some non-linear deviations in position, this technique is widely used due to its speed and the absence of (reliable) position sensors [42].

### 2.3. Characterization Techniques

XRD patterns of the powders were recorded on a Philips X’PERT diffractometer (Amsterdam, The Netherlands) that was equipped with a proportional detector and a secondary graphite monochromator. The data were collected stepwise over the range 2θ = 2–20°, at steps of 0.02°, an accumulation time of 20 s per step, using the Cu Kα radiation (λ = 1.54178 Å).

UV–Vis optical spectroscopy of the perovskite powders was carried out using a Cary 5G spectrophotometer (Santa Clara, CA, USA) and CaSO_4_ as reference.

Field-emission scanning electron microscopy (FESEM) images were recorded with a Zeiss (Jena, Germany) Ultra 55 field FESEM apparatus.

Lifetime photoluminescence measurements on the single crystals were carried out using an inverted microscope, Nikon (Tokyo, Japan) Ti2-U, equipped with an XY motorized stage. The emission signal was transmitted through optical fibers to an Edinburgh Instruments (Livingston, UK) FLS1100 spectrofluorometer, which was coupled to a cooled photomultiplier (PMT-980). The measurements were performed at room temperature, utilizing a 405 nm excitation wavelength provided by a picosecond (ps) laser diode incorporated into the microscope. The lifetimes (τ) were calculated from the best fitting of the signal to a single-exponential decay (I(t) = I(0)exp(−t/τ)).

## 3. Results and Discussion

### 3.1. Crystal Characterization

**Figure 2:**
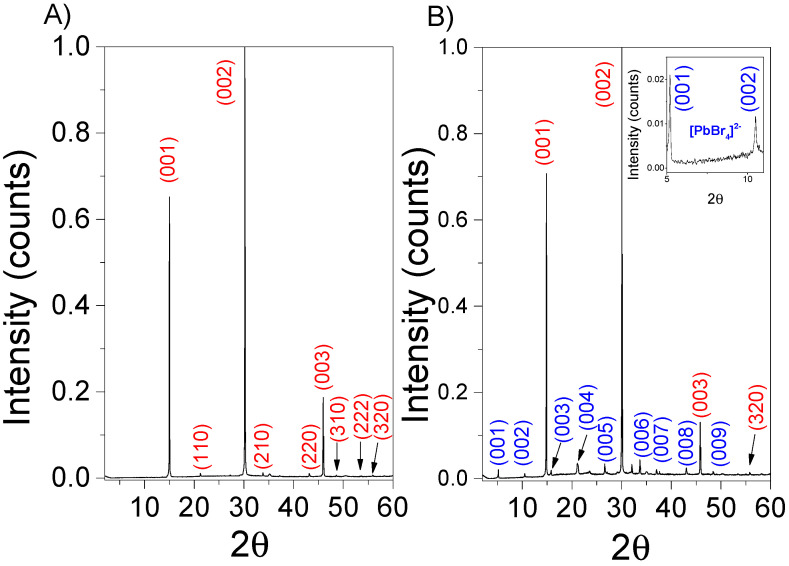
X-ray diffraction patterns for 3D (**A**) and 2D–3D perovskite (**B**). The inset shows a magnification of the low angles region that is characteristic of the layered phase. Red peaks correspond to the 3D phase and blue peaks to the 2D phase (PbBr_4_)^2−^.

The studied single crystals were characterized by XRD. In Figure 2A, the X-ray patterns correspond to the 3D structure perovskite, exhibiting characteristic peaks at 14.77, 29.95, and 45.74 degrees, which are assigned to the (100), (200), and (300) crystal planes, respectively [43,44,45]. This high-intensity XRD diffraction pattern confirms a high phase purity, indicating a preferred orientation in the (001) plane. For the 2D–3D perovskite, additional lower-angle diffraction peaks at 5.10, 10.51, 15.93, 21.17, 26.73, 32.26, 37.7, and 42.9 degrees are observed, corresponding to (001), (002), (003), (004), (005), (006), (007), and (008), respectively. These peaks are characteristic of the 2D phase within these multidimensional 2D–3D structures. Furthermore, they also indicate preferential growth in the (001) plane, suggesting that the 2D phase predominantly grows along the organic or inorganic layer planes [46,47,48,49].

**Figure 3:**
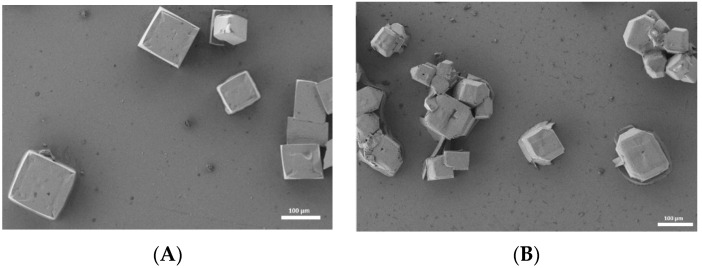
FESEM image of crystalline 3D (**A**) and 2D–3D Perovskite (**B**).

The samples were also characterized by electron microscopy (see Figure 3). In both cases, cubic-shaped crystals with a size of approximately 50–100 µm can be observed. In the case of mixed 2D–3D structure (Figure 3B), the crystals exhibit a more random shape and size distribution, with the formation of lateral heterostructures attributed to the 2D phase. This effect is associated with the presence of the long-chain PEA cation during the crystal growth, which is consistent with previous studies [50].

**Figure 4:**
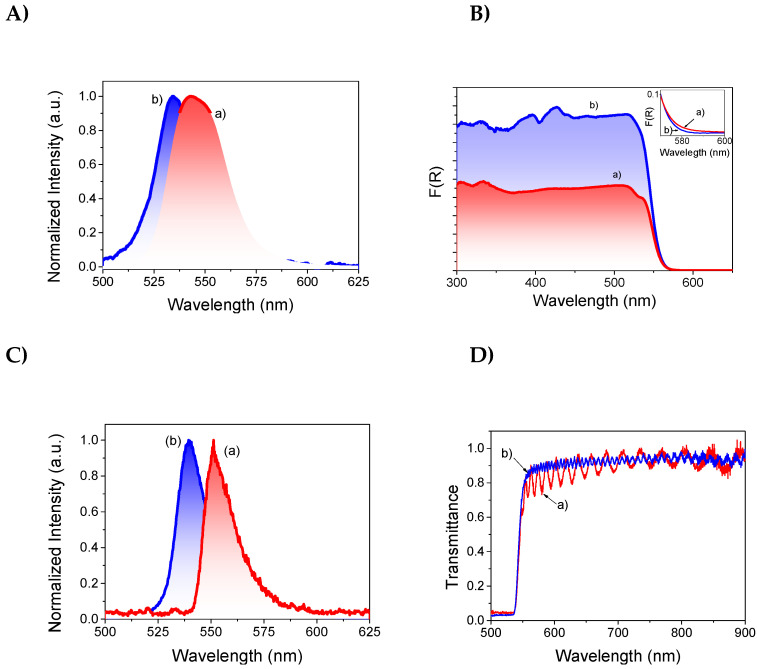
(**A**) Photoluminescence (PL) emission spectra of polycrystalline samples 3D (a) and 2D–3D (b). (**B**) Diffuse reflectance UV–Vis absorption spectra (plotted on the Kubelka–Munk function of the reflectance) F(R) of (a) 3D and (b) mixed 2D–3D perovskite; the inset shows a magnification of the absorption edge of both samples. (**C**) PL emission of single crystals measured with our SCPM and (**D**) transmittance spectrum of the single crystals of (a) 3D and (b) mixed 2D–3D perovskite. They have a thickness of 4 and 13 μm, respectively.

The optical properties of the 3D and 2D–3D perovskites were first obtained by measuring the UV–Vis diffuse reflectance spectra and the photoluminescence in polycrystalline samples. The optical bandgap was calculated to be 2.18 and 2.22 eV for the 3D and 2D–3D, respectively, (Figure S1, ESI) through their corresponding Tauc Plots, which agrees with the previous reported values [49]. It should be noted that the bandgap of the 2D–3D perovskite presents a slight shift at a high energy value due dielectric quantum confinement effects corresponding to the 2D phase (see inset in Figure 4B) [51].

The steady-state luminescence on polycrystalline films shows the characteristic peak emission located at 545 and 535 nm for the 3D and 2D–3D perovskites, respectively (Figure 4A). It should be noted that these samples exhibit a broad emission band compared to the emission observed in selected single crystals, where the emission peaks are well defined (Figure 4C); this is one reason why single crystals are preferred for optoelectronic applications. The broadness of the emission band in polycrystalline films is generally attributed to the scattering of the light by the particles and the defects on the surface and interphases [32]. In addition, comparative temporal profiles of fluorescence decays for both 3D and 2D–3D perovskite single crystals (Figure S5, ESI) allow us to study changes in carrier lifetimes of these perovskites, resulting in values of 9.70 ns for 3D and 18.39 ns for 2D–3D perovskite, respectively. The longer fluorescence lifetime observed in 2D–3D perovskite, compared to 3D perovskite, can be attributed to the reduced nonradiative recombination due to the defect passivation effect of PEABr, which is consistent with previous reports. [52,53]. Nonetheless, and likely due to the limited carrier mobility in a screened layered structure, the prolonged lifetime of 2D–3D perovskite does not lead to greater effective decay length extraction when compared to 3D perovskites, as we will show in point 3.2.

The transmission spectra of the single crystals were also investigated (Figure 4D). The sharp transition from low to high transmittance values corresponds to the band gap and it is in good agreement with diffuse reflectance measurements (Figure 4B). The spectral oscillations in the transparency window are produced by Fabry–Pérot-type optical resonances occurring between opposite crystal faces in the measurement direction. This behavior, known as an optical cavity in hybrid perovskites, has already been reported [54]. We took a step forward by developing a model capable of determining optical properties, such as the refractive index and dielectric constant, as a function of the crystal size, PL and transmission [29,30,55]. According to this model, one of the main factors influencing the amplitude and periodicity of the oscillations associated with optical resonances is the crystal thickness. This way, the 3D crystal, with a thickness (measured by optical profilometry means) of 4 μm gives rise to oscillations with much longer periodicity than those of the 2D crystal, which has a thickness of 13 μm. Deviations in the refractive index between both crystals could additionally influence this phenomenon, but we considered them negligible in comparison with the thickness effect. In any case, the appearance of oscillations in the spectra is a sign of the good quality of the crystals, since they are very sensitive to defects.

### 3.2. Scanning Photocurrent Microscopy

**Figure 5:**
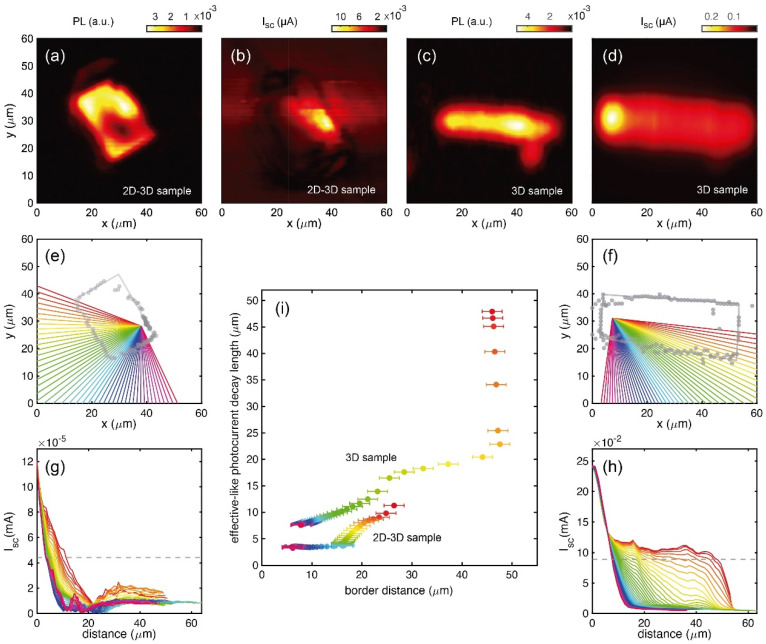
Scanning photocurrent microscopy images and profile analysis. (**a**) PL and (**b**) I_sc_ images of a multidimensional 2D–3D sample. (**c**) PL and (**d**) I_sc_ images of a 3D sample. (**e**,**f**) Edge extraction and (**g**,**h**) photocurrent sections of the 2D–3D and 3D samples, respectively. (**i**) Effective-like photocurrent length extracted from the dashed lines (*e*^−1^) of (**g**,**h**). The different colors correspond to the data (**g**–**i**) extracted from different angular slices (**e**,**f**).

Single perovskite crystals were measured and analyzed with an SPCM technique. Figure 5 shows the photoluminescence (PL) and photocurrent (I_SC_) maps for a multidimensional 2D–3D perovskite crystal (panels (a) and (b)) and for a 3D perovskite crystal (panels (c) and (d)), respectively. The sample limits (panels (e) and (f), corresponding to 2D–3D and 3D samples, respectively) were extracted directly from the image scans. For that purpose, a quadrilateral geometry was fitted to a set of points, extracted by evaluating the derivative of the map signals. Then, we used those maps with best defined sample edges (panels (a) and (d) for the multidimensional 2D–3D and 3D crystals, respectively). Panels (g) and (h) show the photocurrent profiles extracted from the I_SC_ maps through different directions indicated by the colored lines in panels (e) and (f), respectively. The data cover from short (SPED) to long probe-to-edge distances (LPED) and reveal notable differences among the profile curves. Considering that the exponential decay value represents the distance from the collection electrode at which the intensity decays a factor of $e^{-1}\approx 0.368$ of the maximum value, we evaluated the intersection of *e*^−1^ with all the extracted profiles [panels (g) and h] (see dashed horizontal lines). We employed this method for extracting an effective-like photocurrent decay length (ELPD) among complex decay data that could be influenced by several factors such as different material responses, proximity of sample edges and the presence of defects and inhomogeneities. In fact, the ELPD values in panel (i) are represented against the distance from the collecting punctual probe to the sample border in the direction of the corresponding decay profile. This makes it possible to establish an interesting comparative evaluation. In general, the ELPD tendency is to rise with increasing distance to the border, where recombination losses are more prominent. This tendency seems to be modulated by the rest of the surrounding rims, which define the geometry of the sample itself, because the growing tendency of ELPD is flattened at short distances for both the 2D–3D and 3D crystals (pink to light blue dots) and at an intermediate distance for the 3D crystal (yellow dots), and it is boosted at intermediate and long distances for the 2D–3D and 3D crystal, respectively. This flatness/boost tendency of ELPD could be explained, firstly, by the proximity/remoteness of the path to all the external borders, and secondly, by the angle defined between the cross section to the path and the corresponding intersected outer rim, the tangent/normal case being the worst/best scenario for a long ELPD. Interestingly, in the 3D sample, under LPED conditions, a protuberance emerges, creating an almost flat region, resulting in a giant boost. This effect shows a huge difference between samples across all panels of the Figure 5, where the ELPD of the 3D is greater than that of multidimensional 2D–3D, as expected. This may confirm the assumption of the lower photon harvesting efficiency of the 2D–3D in contrast to the 3D counterparts, attributed to the presence of interlayer screening and higher exciton binding energy.

**Figure 6:**
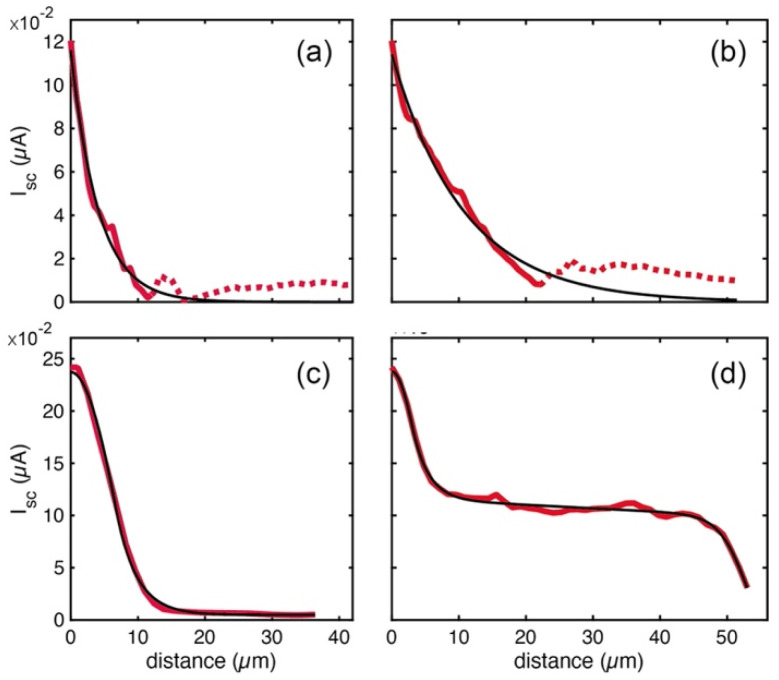
Model fit to experimental photocurrent profiles. (**a**,**b**) Experimental (red curves) and fitted (black curves) profiles for the 2D–3D sample corresponding to a short (STED) and a long (LTED) distance to the collection tip electrode, respectively. The dashed sections indicate experimental points that were disregarded for the fit process. (**c**,**d**) The same as (**a**,**b**) but for the 3D sample.

In order to extract more precise data from the photocurrent measurements and support the previous analysis, two I_SC_ profiles corresponding to SPED and LPED were fitted to phenomenological models for both crystal types. They correspond to pink and red dots in Figure 5i, with edge distances of 6.02 and 26.42 µm for the 2D–3D case (red curves in Figure 6a,b, respectively), and 7.78 and 46.02 µm for the 3D case (red curves in Figure 6c,d, respectively). The fitting model of the 2D–3D sample consists of a single exponential function in the form of $J\propto e^{\frac{-x}{D}}$, where *D* is a fitting decay parameter. The fitted curves (black lines) agree well with the experiment except for very low I_SC_ values that were disregarded (dashed lines), where the signal slightly oscillates due to some unmeaningful factors (e.g., the presence of scattered light). The results show that *L_D_* is clearly longer (in fact, it doubles its magnitude) for the LPED (10.71 ± 0.08 µm) than for the SPED (4.08 ± 0.042 µm) profile, which could be attributed to the large difference in the edge-to-tip distance.

On the other hand, more complex models with additional parameters were required in the 3D case (Figure 6c,d) because the experimental curves (red lines) display richer profiles. Firstly, both SPED and LPED present a smooth peak at zero distance for which a simple Gaussian function that extends from the origin (0 µm) to its half width was used. Secondly, an exponential function accounting for the fast decay of I_SC_ ($J\propto e^{\frac{-x}{D_{1}}}$) was added. Thirdly, a small offset was summed to the SPED profile (Figure 6c), while a second, long exponential decay ($J\propto e^{\frac{-x}{D_{2}}}$) was considered for the LPED profile (Figure 6d). Strictly speaking, such a long decay should be present in all the profiles of the 3D crystal; however, we think it is hidden when the sample borders are close to the collection probe because the borders may deactivate the charge carriers. Finally, another half-Gaussian curve that accounts for the sample edge was added for the LPED profile.

We hypothesize that the initial Gaussian peak at the origin is associated with the electric contact of the probe with the crystal surface. At the interface, the higher metal/perovskite built-in potential allows for easier dissociation of excitons. The fits yielded values for the gaussian half-width of 6.55 ± 0.41 µm and 2.92 ± 0.53 µm for the SPED and LPED profiles, respectively. We attribute the presence/absence or differences in the symmetry of this peak to experimental conditions related to the contact of the electronic probe. Indeed, the peak at the origin is not present in the 2D–3D crystal, likely due to its fragility compared to the 3D perovskite [56,57].

The short exponential decays yielded quite similar D_1_ values for the SPED and LPED profiles (3.06 ± 0.03 µm and 2.35 ± 0.05 µm, respectively), while the large exponential decay for LPED gave a D_2_ value of 328.88 ± 13.73 µm, and the center position of the second Gaussian was fitted at 48.89 µm ± 0.53, which is quite similar to the assumed border distance: 46.02 µm. With regard to the D_1_, in the proximity of both the source and drain contacts, the current could be increased because the photogenerated electrons and holes show the same probability to reach the contacts [35]. On the other hand, the large D_2_ value could be the fingerprint of the higher-efficiency photocurrent harvesting of the 3D perovskite with respect to the multidimensional one. Although the extracted values of decay lengths could not be directly comparable to the diffusion lengths reported in the literature because of the sample and experimental conditions in our scheme, the relationship between the values of D_1_ and D_2_ exhibits significant differences, with the 3D perovskite case clearly showing a larger value, as expected.

## 4. Conclusions

In summary, this study demonstrates the role of the composition of hybrid halide perovskites in their optoelectronic properties, particularly the influence on the spatial dynamics of the photogenerated transport charges along the microcrystals. Two types of single crystals of perovskites were investigated based on the incorporated organic cation: conventional 3D structures and multidimensional 2D–3D structures. A scanning photocurrent microscopy technique based on a probe tip shows different charge distributions over the perovskite single crystals; the 3D perovskites exhibited two distinct carrier transport regimes. In contrast, the transport of carriers in multidimensional 2D–3D structures was primarily dominated by a unique regime. All of them are influenced by the presence of the border as a recombination source. Overall, this study highlights the significance of the dimensionality of perovskite materials in their optoelectronic and transport properties. Furthermore, studying single crystals provides a valuable platform for exploring these fundamental characteristics.

## Data Availability

The data supporting the findings of this study are available from the corresponding authors upon reasonable request.

## Supplementary Materials

The following supporting information can be downloaded at: https://www.mdpi.com/article/10.3390/nano13182570/s1, Figure S1: Tauc plots of 3D and 2D–3D perovskites; Figure S2: Schematic representation of the homemade SPCM used; Figure S3: Optical microscope images of single crystals of 3D and 2D–3D perovskites; Figure S4: Optical microscope image showing the electronic contact on a single crystal; Figure S5: PL decays lifetime of single crystals of 3D and 2D–3D multidimensional perovskites; Figure S6: Photography of the antisolvent system used for the growth of the single crystals.

## References

[1] Bati A.S.R., Zhong Y.L., Burn P.L., Nazeeruddin M.K., Shaw P.E., Batmunkh M. (2023). Next-generation applications for integrated perovskite solar cells. Commun. Mater..

[2] Kieslich G., Sun S., Cheetham A.K. (2015). An extended Tolerance Factor approach for organic–inorganic perovskites. Chem. Sci..

[3] Liu Y., Guo W., Hua L., Zeng X., Yang T., Fan Q., Ma Y., Gao C., Sun Z., Luo J. (2023). Giant Polarization Sensitivity via the Anomalous Photovoltaic Effect in a Two-Dimensional Perovskite Ferroelectric. J. Am. Chem. Soc..

[4] Han S., Li L., Ji C., Liu X., Wang G.-E., Xu G., Sun Z., Luo J. (2023). Visible-Photoactive Perovskite Ferroelectric-Driven Self-Powered Gas Detection. J. Am. Chem. Soc..

[5] Pham P.V., Bodepudi S.C., Shehzad K., Liu Y., Xu Y., Yu B., Duan X. (2022). 2D Heterostructures for Ubiquitous Electronics and Optoelectronics: Principles, Opportunities, and Challenges. Chem. Rev..

[6] Mao L., Stoumpos C.C., Kanatzidis M.G. (2019). Two-Dimensional Hybrid Halide Perovskites: Principles and Promises. J. Am. Chem. Soc..

[7] Mitzi D.B., Chondroudis K., Kagan C.R. (2001). Organic-inorganic electronics. IBM J. Res. Dev..

[8] Frohna K., Stranks S.D., Ostroverkhova O. (2019). 7–Hybrid Perovskites for Device Applications. Handbook of Organic Materials for Electronic and Photonic Devices.

[9] Sherkar T.S., Momblona C., Gil-Escrig L., Ávila J., Sessolo M., Bolink H.J., Koster L.J.A. (2017). Recombination in Perovskite Solar Cells: Significance of Grain Boundaries, Interface Traps, and Defect Ions. ACS Energy Lett..

[10] Shrestha S., Li X., Tsai H., Hou C.-H., Huang H.-H., Ghosh D., Shyue J.-J., Wang L., Tretiak S., Ma X. (2022). Long carrier diffusion length in two-dimensional lead halide perovskite single crystals. Chem.

[11] Turedi B., Lintangpradipto M.N., Sandberg O.J., Yazmaciyan A., Matt G.J., Alsalloum A.Y., Almasabi K., Sakhatskyi K., Yakunin S., Zheng X. (2022). Single-Crystal Perovskite Solar Cells Exhibit Close to Half A Millimeter Electron-Diffusion Length. Adv. Mater..

[12] Tian W., Zhao C., Leng J., Cui R., Jin S. (2015). Visualizing Carrier Diffusion in Individual Single-Crystal Organolead Halide Perovskite Nanowires and Nanoplates. J. Am. Chem. Soc..

[13] Misra R.K., Ciammaruchi L., Aharon S., Mogilyansky D., Etgar L., Visoly-Fisher I., Katz E.A. (2016). Effect of Halide Composition on the Photochemical Stability of Perovskite Photovoltaic Materials. ChemSusChem.

[14] Guo Z., Wan Y., Yang M., Snaider J., Zhu K., Huang L. (2017). Long-range hot-carrier transport in hybrid perovskites visualized by ultrafast microscopy. Science.

[15] Yang Y., Ostrowski D.P., France R.M., Zhu K., van de Lagemaat J., Luther J.M., Beard M.C. (2016). Observation of a hot-phonon bottleneck in lead-iodide perovskites. Nat. Photonics.

[16] D’innocenzo V., Grancini G., Alcocer M.J.P., Kandada A.R.S., Stranks S.D., Lee M.M., Lanzani G., Snaith H.J., Petrozza A. (2014). Excitons versus free charges in organo-lead tri-halide perovskites. Nat. Commun..

[17] Lang D.V., Henry C.H. (1978). Scanning photocurrent microscopy: A new technique to study inhomogeneously distributed recombination centers in semiconductors. Solid-State Electron..

[18] O’dea J.R., Brown L.M., Hoepker N., Marohn J.A., Sadewasser S. (2012). Scanning probe microscopy of solar cells: From inorganic thin films to organic photovoltaics. MRS Bull..

[19] Hao Y., He C., Xu J., Bao Y., Wang H., Li J., Luo H., An M., Zhang M., Zhang Q. (2022). High-Performance van der Waals Photodetectors Based on 2D Ruddlesden–Popper Perovskite/MoS_2_ Heterojunctions. J. Phys. Chem. C.

[20] Ha D., Yoon Y., Park I.J., Cantu L.T., Martinez A., Zhitenev N. (2023). Nanoscale Characterization of Photocurrent and Photovoltage in Polycrystalline Solar Cells. J. Phys. Chem. C.

[21] Xu J., Li J., Wang H., He C., Li J., Bao Y., Tang H., Luo H., Liu X., Yang Y. (2022). A Vertical PN Diode Constructed of MoS_2_ /CsPbBr_3_ Heterostructure for High-Performance Optoelectronics. Adv. Mater. Interfaces.

[22] Fang F., Wan Y., Li H., Fang S., Huang F., Zhou B., Jiang K., Tung V., Li L.-J., Shi Y. (2022). Two-Dimensional Cs_2_AgBiBr_6_/WS_2_ Heterostructure-Based Photodetector with Boosted Detectivity via Interfacial Engineering. ACS Nano.

[23] Wen X., Jia B. (2022). New insight into carrier transport in 2D layered perovskites. Chem.

[24] Ahn Y., Dunning J., Park J. (2005). Scanning Photocurrent Imaging and Electronic Band Studies in Silicon Nanowire Field Effect Transistors. Nano Lett..

[25] Leite M.S., Abashin M., Lezec H.J., Gianfrancesco A., Talin A.A., Zhitenev N.B. (2014). Nanoscale Imaging of Photocurrent and Efficiency in CdTe Solar Cells. ACS Nano.

[26] Otto T., Miller C., Tolentino J., Liu Y., Law M., Yu D. (2013). Gate-Dependent Carrier Diffusion Length in Lead Selenide Quantum Dot Field-Effect Transistors. Nano Lett..

[27] Lombardo C.J., Glaz M.S., Ooi Z.-E., Vanden Bout D.A., Dodabalapur A. (2012). Scanning photocurrent microscopy of lateral organic bulk heterojunctions. Phys. Chem. Chem. Phys..

[28] Graham R., Yu D. (2013). Scanning photocurrent microscopy in semiconductor nanostructures. Mod. Phys. Lett. B.

[29] Ramiro-Manzano F., García-Aboal R., Fenollosa R., Biasi S., Rodriguez I., Atienzar P., Meseguer F. (2020). Optical properties of organic/inorganic perovskite microcrystals through the characterization of Fabry–Pérot resonances. Dalton Trans..

[30] Segura-Sanchis E., Fenollosa R., Rodriguez I., Molard Y., Cordier S., Feliz M., Atienzar P. (2022). Octahedral Molybdenum Cluster-Based Single Crystals as Fabry–Pérot Microresonators. Cryst. Growth Des..

[31] Rodriguez I., Fenollosa R., Ramiro-Manzano F., García-Aboal R., Atienzar P., Meseguer F.J. (2019). Groove-assisted solution growth of lead bromide perovskite aligned nanowires: A simple method towards photoluminescent materials with guiding light properties. Mater. Chem. Front..

[32] García-Aboal R., García H., Remiro-Buenamañana S., Atienzar P. (2021). Expanding the photoresponse of multidimensional hybrid lead bromide perovskites into the visible region by incorporation of subphthalocyanine. Dalton Trans..

[33] Lou Y., Zhang S., Gu Z., Wang N., Wang S., Zhang Y., Song Y. (2023). Perovskite single crystals: Dimensional control, optoelectronic properties, and applications. Mater. Today.

[34] Rong S.-S., Faheem M.B., Li Y.-B. (2021). Perovskite single crystals: Synthesis, properties, and applications. J. Electron. Sci. Technol..

[35] Xiao R., Hou Y., Fu Y., Peng X., Wang Q., Gonzalez E., Jin S., Yu D. (2016). Photocurrent Mapping in Single-Crystal Methylammonium Lead Iodide Perovskite Nanostructures. Nano Lett..

[36] Yang B., Chen J., Shi Q., Wang Z., Gerhard M., Dobrovolsky A., Scheblykin I.G., Karki K.J., Han K., Pullerits T. (2018). High Resolution Mapping of Two-Photon Excited Photocurrent in Perovskite Microplate Photodetector. J. Phys. Chem. Lett..

[37] Ganesh N., Ghorai A., Krishnamurthy S., Banerjee S., Narasimhan K.L., Ogale S.B., Narayan K.S. (2020). Impact of trap filling on carrier diffusion in MAPbBr_3_ single crystals. Phys. Rev. Mater..

[38] Zhang F., Yang B., Li Y., Deng W., He R. (2017). Extra long electron–hole diffusion lengths in CH_3_NH_3_PbI_3−x_Cl_x_ perovskite single crystals. J. Mater. Chem. C.

[39] Fu D., Zou J., Wang K., Zhang R., Yu D., Wu J. (2011). Electrothermal Dynamics of Semiconductor Nanowires under Local Carrier Modulation. Nano Lett..

[40] Wei Y.-C., Chu C.-H., Mao M.-H. (2021). Minority carrier decay length extraction from scanning photocurrent profiles in two-dimensional carrier transport structures. Sci. Rep..

[41] Teng P., Reichert S., Xu W., Yang S.-C., Fu F., Zou Y., Yin C., Bao C., Karlsson M., Liu X. (2021). Degradation and self-repairing in perovskite light-emitting diodes. Matter.

[42] Huang B., Clark G., Navarro-Moratalla E., Klein D.R., Cheng R., Seyler K.L., Zhong D., Schmidgall E., McGuire M.A., Cobden D.H. (2017). Layer-dependent ferromagnetism in a van der Waals crystal down to the monolayer limit. Nature.

[43] Tisdale J.T., Smith T., Salasin J.R., Ahmadi M., Johnson N., Ievlev A.V., Koehler M., Rawn C.J., Lukosi E., Hu B. (2018). Precursor purity effects on solution-based growth of MAPbBr_3_ single crystals towards efficient radiation sensing. CrystEngComm.

[44] Peng W., Wang L., Murali B., Ho K.-T., Bera A., Cho N., Kang C.-F., Burlakov V.M., Pan J., Sinatra L. (2016). Solution-Grown Monocrystalline Hybrid Perovskite Films for Hole-Transporter-Free Solar Cells. Adv. Mater..

[45] Shen H., Nan R., Jian Z., Li X. (2019). Defect step controlled growth of perovskite MAPbBr_3_ single crystal. J. Mater. Sci..

[46] Di J., Li H., Chen L., Zhang S., Hu Y., Sun K., Peng B., Su J., Zhao X., Fan Y. (2022). Low Trap Density Para-F Substituted 2D PEA_2_PbX_4_ (X = Cl, Br, I) Single Crystals with Tunable Optoelectrical Properties and High Sensitive X-ray Detector Performance. Research.

[47] Dhanabalan B., Leng Y.-C., Biffi G., Lin M.-L., Tan P.-H., Infante I., Manna L., Arciniegas M.P., Krahne R. (2020). Directional Anisotropy of the Vibrational Modes in 2D-Layered Perovskites. ACS Nano.

[48] Tien C.-H., Lee K.-L., Tao C.-C., Lin Z.-Q., Lin Z.-H., Chen L.-C. (2022). Two-Dimensional (PEA)_2_PbBr_4_ Perovskites Sensors for Highly Sensitive Ethanol Vapor Detection. Sensors.

[49] Cohen B.-E., Wierzbowska M., Etgar L. (2017). High Efficiency and High Open Circuit Voltage in Quasi 2D Perovskite Based Solar Cells. Adv. Funct. Mater..

[50] Yang T., Li F., Lin C.-H., Guan X., Yao Y., Yang X., Wu T., Zheng R. (2023). One-pot solution synthesis of 2D-3D mixed-dimensional perovskite crystalline lateral heterostructures. Cell Rep. Phys. Sci..

[51] Jiang Y., Yuan J., Ni Y., Yang J., Wang Y., Jiu T., Yuan M., Chen J. (2018). Reduced-Dimensional α-CsPbX_3_ Perovskites for Efficient and Stable Photovoltaics. Joule.

[52] Li S., Hu L., Zhang C., Wu Y., Liu Y., Sun Q., Cui Y., Hao Y., Wu Y. (2020). In situ growth of a 2D/3D mixed perovskite interface layer by seed-mediated and solvent-assisted Ostwald ripening for stable and efficient photovoltaics. J. Mater. Chem. C.

[53] Yoo H.-S., Park N.-G. (2018). Post-treatment of perovskite film with phenylalkylammonium iodide for hysteresis-less perovskite solar cells. Sol. Energy Mater. Sol. Cells.

[54] Zhang Y., Lim C.-K., Dai Z., Yu G., Haus J.W., Zhang H., Prasad P.N. (2019). Photonics and optoelectronics using nano-structured hybrid perovskite media and their optical cavities. Phys. Rep..

[55] García-Aboal R., Fenollosa R., Ramiro-Manzano F., Rodríguez I., Meseguer F., Atienzar P. (2018). Single Crystal Growth of Hybrid Lead Bromide Perovskites Using a Spin-Coating Method. ACS Omega.

[56] Kim D., Vasileiadou E.S., Spanopoulos I., Kanatzidis M.G., Tu Q. (2021). In-Plane Mechanical Properties of Two-Dimensional Hybrid Organic–Inorganic Perovskite Nanosheets: Structure–Property Relationships. ACS Appl. Mater. Interfaces.

[57] Rathore S., Leong W.L., Singh A. (2023). Mechanical properties estimation of 2D–3D mixed organic-inorganic perovskites based on methylammonium and phenylethyl-ammonium system using a combined experimental and first-principles approach. J. Alloys Compd..

