# Supplementary material for: Scanning Photocurrent Microscopy in Single Crystal Multidimensional Hybrid Lead Bromide Perovskites
Source: Nanomaterials (Basel). 2023 Sep 16;13(18):2570. doi: 10.3390/nano13182570 (PMC10535732; doi:10.3390/nano13182570)
Supplement: Supplementary file 1 [file nanomaterials-13-02570-s001.zip › nanomaterials-2573851-supplementary.pdf]

Supplementary information

Scanning Photocurrent Microscopy in Single Crystal Multidimensional Hybrid Lead Bromide Perovskites

Elena Segura-Sanchis, Rocío García-Aboal, Roberto Fenollosa, Fernando Ramiro-Manzano * and Pedro Atienzar *

Instituto de Tecnología Química, Consejo Superior de Investigaciones Científicas, Universitat Politècnica de València, Avenida de los Naranjos s/n, 46022 Valencia, Spain; elsesan@itq.upv.es (E.S.-S.); rociogarciaaboal@gmail.com (R.G.-A.); rfenollo@ter.upv.es (R.F.)

* Correspondence: ferraman@fis.upv.es (F.R.-M.); pedatcor@itq.upv.es (P.A.)

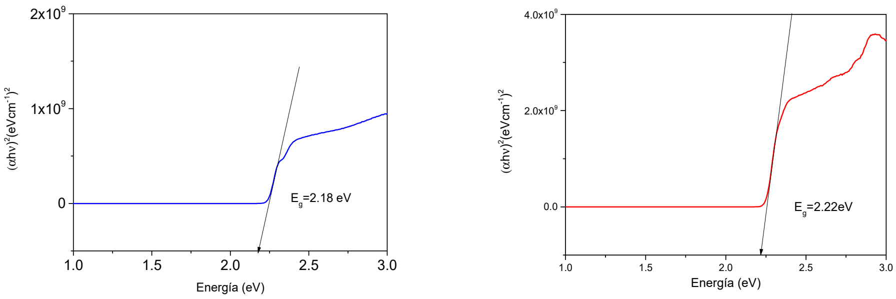

Figure S1. Tauc plot of $(\alpha h\nu)^{0.5}$ vs. photon energy (hu) for the 3D perovskite (left) and 2D-3D (right). The optical band gap of semiconductor can be estimated from the intercept of the extrapolated linear fit for the plotted experimental data of (αhν)n versus incident photon energy (hν) near the absorption edge.

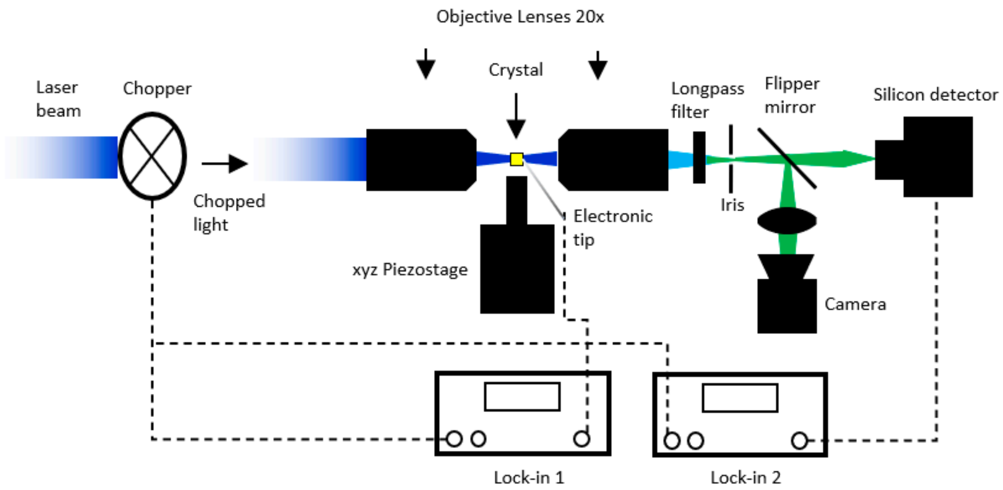

Figure S2. Schematic representation of the homemade SPCM used.

A) B)

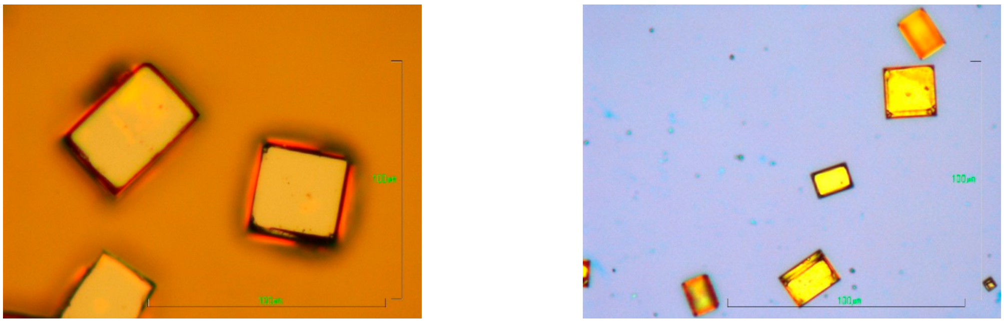

Figure S3. Optical microscope images of single crystals of 3D (A) and 2D-3D multidimensional perovskites (B) deposited on top of a conductive indium tin oxide substrate (ITO).

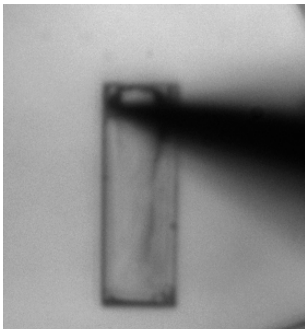

Figure S4. Optical microscopy image showing a single crystal on a conductive substrate, with the electronic tip making contact on the top surface.

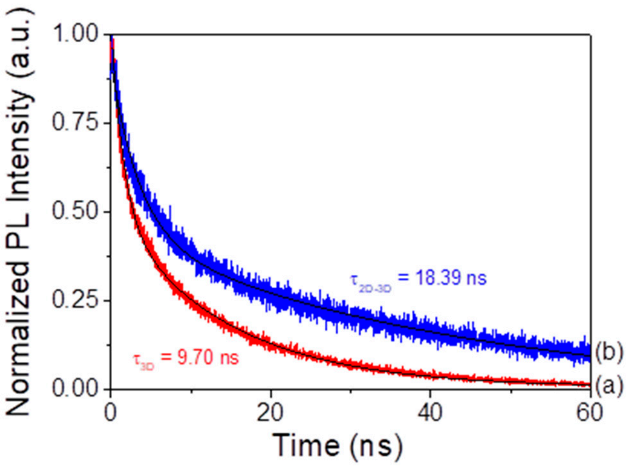

Figure S5. PL decays lifetime of single crystals of 3D (a) and 2D-3D multidimensional perovskites (b) measured at 545 and 535 nm respectively.

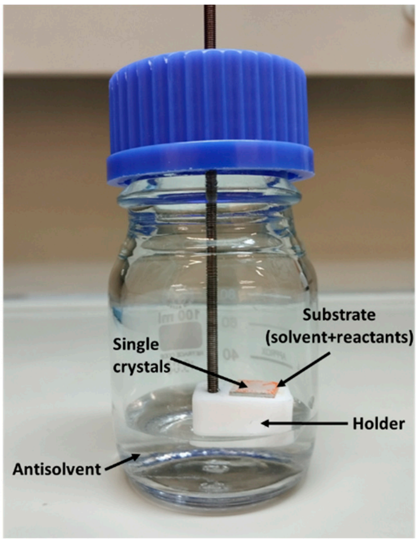

Figure S6. Photography of the antisolvent system used for the growth of the single crystals.
